# The role of manual gestures in second language comprehension: a simultaneous interpreting experiment

**DOI:** 10.3389/fpsyg.2023.1188628

**Published:** 2023-06-27

**Authors:** Eléonore Arbona, Kilian G. Seeber, Marianne Gullberg

**Affiliations:** ^1^Faculty of Translation and Interpreting, University of Geneva, Geneva, Switzerland; ^2^Centre for Languages and Literature and Lund University Humanities Lab, Lund University, Lund, Sweden

**Keywords:** manual gestures, simultaneous interpreting, second language comprehension, language comprehension, integrated-systems hypothesis

## Abstract

Manual gestures and speech form a single integrated system during native language comprehension. However, it remains unclear whether this hold for second language (L2) comprehension, more specifically for simultaneous interpreting (SI), which involves comprehension in one language and simultaneous production in another. In a combined mismatch and priming paradigm, we presented Swedish speakers fluent in L2 English with multimodal stimuli in which speech was congruent or incongruent with a gesture. A picture prime was displayed before the stimuli. Participants had to decide whether the video was related to the prime, focusing either on the auditory or the visual information. Participants performed the task either during passive viewing or during SI into their L1 Swedish (order counterbalanced). Incongruent stimuli yielded longer reaction times than congruent stimuli, during both viewing and interpreting. Visual and audio targets were processed equally easily in both activities. However, in both activities incongruent speech was more disruptive for gesture processing than incongruent gesture was for speech processing. Thus, the data only partly supports the expected mutual and obligatory interaction of gesture and speech in L2 comprehension. Interestingly, there were no differences between activities suggesting that the language comprehension component in SI shares features with other (L2) comprehension tasks.

## 1. Introduction

We have long known that we gesture when we speak. It has been suggested that manual co-speech gestures and speech in fact are intimately related, forming an integrated system (McNeill, 1992; Kendon, 2004). In other words, gestures performed in association with speech “are produced as an integral part of the same plan of action as the spoken utterance” (Kendon, 1994, p. 176). Importantly, gestures have semantic functions (e.g., drawing a shape in the air to add detail to accompanying speech) and pragmatic functions (e.g., a “brushing aside” gesture to dismiss a possibility mentioned in speech) that parallel those of speech (McNeill, 1985). Gesture articulation is also synchronized with linguistic units in speech (Kendon, 2004; see McNeill, 1992 for an in-depth discussion of the growth point hypothesis which specifically addresses the synchronization issue). For example, energetic contrasts in gesture (e.g., strokes) are structurally related to prosodic contrasts, e.g., peak pitch or stressed syllable (Pouw and Dixon, 2019). Temporal and semantic co-expressivity of gesture and speech has been convincingly demonstrated both in L1 and in L2 populations. For instance, Gullberg et al. (2008) showed that L1 speakers were overwhelmingly co-expressive in speech and gesture: speech temporally aligned with gestures expressed the same semantic meaning, e.g., path gestures co-occurred mainly with path verbs. Gullberg (2009) tested L1 English speakers of L2 Dutch on verbs of placement, investigating the potential effect of switching from a single-term system (English) to a multiple-term system (Dutch). She found that the more L2 speakers produced gestures expressing a L2-like focus on objects, the more likely they were to appropriately use placement verbs in speech in the L2. In other words, in L2 speakers, overall, gesture and speech were co-expressive, and synchronized both temporally and semantically. Finally, studies show that gesture develops together with speech in children (Goldin-Meadow, 1998; Mayberry and Nicoladis, 2000; Capirci and Volterra, 2008; Colletta et al., 2015) and break down at the same time (Mayberry and Jaques, 2000; Rose, 2006; Buck and Powers, 2013; Graziano and Gullberg, 2018).

In language comprehension, similar parallelisms are found. Generally, gesture and speech are processed in parallel (Holle and Gunter, 2007; Wu and Coulson, 2007; see Kelly, 2017, for an overview). For example, comprehenders have been shown to build a single unified representation of utterances, as they process the information from both channels without necessarily realising which particular channel it came from Cassell et al. (1999), Goldin-Meadow (2003), and Gullberg and Kita (2009). Finally, studies investigating the neural mechanisms of speech-gesture integration have demonstrated that the semantic processing of gesture and speech rely on overlapping resources in the brain (see Özyürek, 2014, for a review).

The nature of the integration in comprehension has been further explored, and specifically the ways in which information in either modality influences the processing of the other modality. Priming studies using an incongruence paradigm have revealed a bidirectional influence of gesture on speech and vice versa (Kelly et al., 2009, 2015). In these studies, first language (L1) English comprehenders were presented with multimodal stimuli in which speech was congruent or incongruent with a gesture. A picture prime was displayed before the stimuli. Comprehenders had to decide whether the video was related to the prime or not, focusing either on the auditory and/or on the visual information. The authors investigated response accuracy and reaction times (RTs) in the identification of the two types of targets (audio and visual) and the extent to which incongruities between speech and gesture disrupted processing. The extent to which comprehenders are unable to ignore irrelevant information within gesture-speech stimuli is considered a measure of the relative strength of their integration (Kelly et al., 2009). The authors showed that when gesture and speech convey the same information, comprehenders answer faster and make fewer errors, compared to when they convey different information. Incongruent gestures disrupted language comprehension to the same extent as incongruent speech, even when one modality was completely irrelevant to the experimental task. This means that comprehenders could not avoid processing both modalities even if focused only on one. The Integrated-Systems Hypothesis, which was developed on this empirical basis, posits that speech and gesture are tightly integrated and mutually and obligatorily interact in order to enhance language comprehension (Kelly et al., 2009). In other words, gestures necessarily influence the processing (i.e., comprehension) of speech, and speech necessarily influences the processing (i.e., comprehension) of gesture.

Özer and Göksun (2020) replicated and extended these results by testing L1 Turkish comprehenders. The authors found lower accuracy and longer RTs in incongruent conditions compared to the congruent baseline, as in Kelly et al. (2009), suggesting that speech-gesture pairs conveying the same information are easier to process than pairs conveying incongruent information. However, comprehenders were slower and less accurate when presented with incongruent *gestural* information compared to with incongruent speech, contrary to Kelly et al. (2009) who found slower RTs (but not higher error rates) for incongruent *speech* compared to incongruent gestures. To explain these differences, the authors mentioned the different sample sizes, stimuli, languages and cultures, as well as the isomorphism between the action primes and the gestures (i.e., the similarity between primes and gestures may have influenced the results).

However, it is not clear whether what applies to first language comprehension is also valid in second language (L2) comprehension. Kelly and colleagues had L1 English comprehenders take part in both studies, which were administered in English, and Özer and Göksun tested L1 Turkish comprehenders for their study in Turkish. Perniss et al. (2020) replicated and extended Kelly et al. (2009) using picture primes, and tested both L1 and advanced L2 comprehenders of English. The L2 comprehenders rated their proficiency in English as 4.03 on a range of 1–5 (with 1 = “not very good” and 5 = “near native”; only participants that rated their English proficiency as 3 or higher were included in the experiment), and their age of first exposure to English was 9.6 on average (range 1–23). In one of the experiments (experiment 3), L1 comprehenders were presented with speech-gesture pairs and were asked to judge whether the speech in the video matched the picture prime. Although comprehenders were less accurate with incongruent speech-gesture pairs than with congruent pairs, in line with Kelly et al. (2009), they responded more slowly to congruent speech-gesture pairs compared to incongruent pairs, in contrast to the original study. In a follow-up experiment (experiment 4), L1 and L2 comprehenders were presented with speech-gesture pairs and had to decide whether any part of the video (speech and/or gesture) matched the picture prime. Perniss and colleagues found that both incongruent speech and incongruent gesture had comparable interfering effects on L1 accuracy (in line with Kelly et al., 2009) and L2 accuracy. Interestingly, in L2 comprehenders, incongruent gestures were more disruptive than incongruent speech. The authors concluded that L2 comprehenders may rely more on gestural information than L1 comprehenders. Moreover, incongruent speech-gesture pairs led to slower RTs in both groups compared to congruent pairs, further replicating the results of Kelly et al. (2009). Taken together, these results suggest that advanced L2 comprehenders are sensitive to gestures during L2 comprehension, perhaps even more so than L1 comprehenders.

More broadly, the literature provides mixed results regarding whether L2 comprehenders use gestures similarly to L1 comprehenders. In Dahl and Ludvigsen (2014), teenage L2 learners of English as a foreign language showed language comprehension patterns comparable to L1 comprehenders when they could access gestures. However, even with access to gestures content distortions were significantly more frequent in L2 comprehenders than in L1 comprehenders. In Sueyoshi and Hardison (2005), low proficiency and high proficiency L2 learners of English were presented with clips with auditory speech, lip movements and gestures, auditory speech and lip movements only, or auditory speech only. The higher proficiency comprehenders benefited most from the auditory speech and lip movements condition whereas the clips with auditory speech, lip movements and gestures yielded the best scores for the lower proficiency comprehenders. This suggests that advanced L2 comprehenders perform best when given access to auditory speech combined with lip movements, and do not necessarily benefit from parallel access to gestures. However, there was no baseline (e.g., L1 comprehenders) to compare the learners to, which makes it difficult to predict whether L1 and L2 comprehenders behave similarly when presented with gestures. Drijvers et al. (2019) showed a clear gestural enhancement effect in both L1 and L2 comprehenders (of upper intermediate level). However, L2 comprehenders gazed more at gestural information than L1 comprehenders. In an EEG study (Drijvers and Özyürek, 2018) investigating the N400 component (thought to be indicative of integration), highly proficient L2 Dutch comprehenders and L1 Dutch comprehenders were presented with multimodal stimuli. The results showed a larger N400 effect in clear speech for L2 comprehenders compared to L1 comprehenders. The authors argued that in clear speech, L2 comprehenders may recruit the visual semantic information more than natives, paying more attention to gestures. Moreover, no N400 effect was found in L2 comprehenders in degraded speech (whereas there was one in L1 comprehenders). The authors concluded that L2 comprehenders benefitted less from gestures in degraded speech, and integrated speech and gesture differently compared to L1 comprehenders. Kang et al. (2013) probed information uptake from scientific discourse including gesture and speech in L1 and (advanced) L2 participants, and found that when speech was accompanied with redundant representational gestures, L2 comprehension scores drastically increased (which was not the case in L1 participants), suggesting that L2 comprehenders relied more on representational gestures than natives and may have been able to compensate for difficult passages in speech. This brief review shows that while advanced L2 comprehenders can be predicted to behave similarly to L1 comprehenders, previous studies have revealed differences in attention to and processing/integration of gestures.

The present study specifically investigates simultaneous interpreting (SI), an extreme instance of L2 use (Hervais-Adelman et al., 2015) involving concurrent comprehension and production in two distinct languages. Traditionally, translation and interpreting studies have focused on written and oral texts, disregarding non-verbal resources (González, 2014), and SI has been considered and modeled primarily as a verbal (oral) task (Seeber, 2017). Consequently, influential models of SI from Gerver (1976) to Setton (1999) focus on the verbal (oral) modality only. However, an emerging line of research investigates SI as a multimodal task (Galvão and Galhano Rodrigues, 2010; Seeber, 2011; Stachowiak-Szymczak, 2019). Indeed, in typical settings, interpreters process speakers’ input while having access to various sources of visual information, including manual gestures (Galvão, 2013; Seeber, 2017; Gieshoff, 2018). Interpreters generally deem visual access necessary for successful interpretation (Bühler, 1985), and manual gestures and facial expressions are considered useful for language comprehension (Rennert, 2008). Visual access to speakers, including to their gestures, is enshrined in the working conditions issued by the International Association of Conference Interpreters (AIIC, 2007) and in ISO standards (International Organization for Standardization, 2016, 2017). Finally, it has been shown that interpreters attend to and benefit from speakers’ gestures during passive viewing/listening and during simultaneous interpreting (Arbona et al., 2023). However, the question whether the Integrated-Systems Hypothesis also applies to SI and whether the modalities carry equal weight for comprehension has not yet been investigated. Crucially, the incongruence paradigm introduced by Kelly et al. (2009) enables us to probe *mutual* and *obligatory* interactions of gesture and speech, which goes beyond simply looking at potential facilitating effects of gestures in language comprehension.

The aim therefore is to shed more light on the relationship between gesture and speech in a SI context, as a specific instance of L2 comprehension. The study will enhance our knowledge of whether and how gesture and speech interact with each other, and contribute to answer the question whether the language comprehension component in SI shares common features with other (L2) comprehension tasks. If the Integrated-Systems Hypothesis applies to SI, this would also inform theoretical development in interpreting studies. More specifically, we had two goals for this study. First, we set out to replicate previous research showing that incongruent multimodal utterances are harder to process than congruent ones and extend it to L2 speakers, especially in a simultaneous interpreting context. Second, we wanted to test two predictions: if the Integrated-Systems Hypothesis also applies to L2 comprehension, more specifically to simultaneous interpreting, participants engaged in L2 comprehension should (1) process audio targets as easily as visual targets and (2) have comparable difficulty ignoring, on the one hand, irrelevant gestures when processing speech and, on the other hand, irrelevant speech when processing gestures. We emphasise that in the current study, language processing is investigated from a *comprehension* perspective only; although language processing includes language production, processing from a *production* angle is beyond the scope of this piece of work.

In the current study, we used a paradigm similar to that of Kelly et al. (2009, 2015) to investigate the Integrated-Systems Hypothesis in L2 comprehension, more specifically in a simultaneous interpreting context. Participants were presented with multimodal stimuli in which speech was congruent or incongruent with a gesture. A picture prime was displayed before the stimuli. Participants had to decide whether the video was related to the prime or not, focusing either on the auditory or the visual information. Participants performed the task in two activities: passive viewing vs. SI. As in Kelly et al. (2009, 2015), we investigated (1) response accuracy and reaction time for identifying the two types of targets (audio and visual) and (2) the extent to which incongruities between speech and gesture disrupted processing.

## 2. Materials and methods

### 2.1. Participants

Forty-eight Swedish university undergraduates (25 males) participated in the experiment, see Table 1. Their L1 was Swedish and L2 was English. Three participants had learnt another language before Swedish (2 participants; they reported Swedish acquisition at 5 years of age and Swedish fluency at 6 years of age) or in parallel with Swedish (1 participant, who reported Swedish acquisition at 1 year of age and Swedish fluency at 3 years of age). Participants were recruited from the Prolific recruitment service^1^ and were redirected to the Gorilla platform^2^ to complete the study. No prior interpreting experience was necessary to take part. Participants completed an adapted version of the Language Experience and Proficiency Questionnaire (Marian et al., 2007). They also completed the English LexTALE test (Lemhöfer and Broersma, 2012) to assess proficiency, which was administered on the Gorilla platform. This test, which provides a good measure of English vocabulary knowledge of medium- to high-proficient learners of English as a second language, has been shown to correlate with measures of English language proficiency.

**Table 1:** Background information provided in the language background questionnaire, and comparison of groups (*t*-test for numerical variables, Wilcoxon test for ordinal variables): *p < 0.05, **p < 0.01, ***p < 0.001.

	Audio (*n* = 24, 12 females)		Visual (*n* = 24, 11 females)		Comparison
	*M*	*SD*	*M*	*SD*	
Background					
Age (yrs)	28.8	8.0	29.7	8.8	*ns*
Languages spoken	3.0	1.0	3.2	1.1	*ns*
Swedish					
Age of acquisition (yrs)	1.8	1.4	1.6	1.4	*ns*
Age of fluency (yrs)	2.5	1.7	3.4	2.1	*ns*
Duration of exposure^1^ (yrs)	25.5	7.7	25.5	8.1	*ns*
Current exposure (%)	53.3	24.9	45.2	26.2	*ns*
English					
Age of acquisition (yrs)	8.0	3.6	8.2	2.8	*ns*
Age of fluency (yrs)	13.7	4.5	15.2	4.6	*ns*
Duration of exposure (yrs)	2.7	5.3	6.2	9.0	*ns*
Current exposure (%)	39.2	24.4	48.8	26.8	*ns*
Self-rated English proficiency					
Speaking	8.3	1.3	8.4	1.1	*ns*
Reading	8.8	1.1	9.2	0.8	*ns*
Listening	8.9	1.0	9.2	0.9	*ns*
LexTALE score (%)	88.6	9.7	88.0	9.2	*ns*

^1^Number of years participants spent in a country or region where the relevant language is spoken.

Participants were proficient in English. Their average LexTALE score was 88.3% (*SD* = 9.4, range: 61.25–100). Scores between 80 and 100% are considered to correspond to advanced learners. Participants started acquiring English at the average age of 8 years (*SD* = 3) and became fluent at the average age of 15 years (*SD* = 5). They reported their current exposure to English as being on average 44% of the time (*SD* = 25.8) and rated their English listening proficiency as 9 (*SD* = 1) out of 10.

All participants gave written informed consent. The experiment was approved by the Faculty of Translation and Interpreting’s Ethics Committee at the University of Geneva. No participant was involved in the norming of the stimuli.

### 2.2. Materials

**Figure 1:**
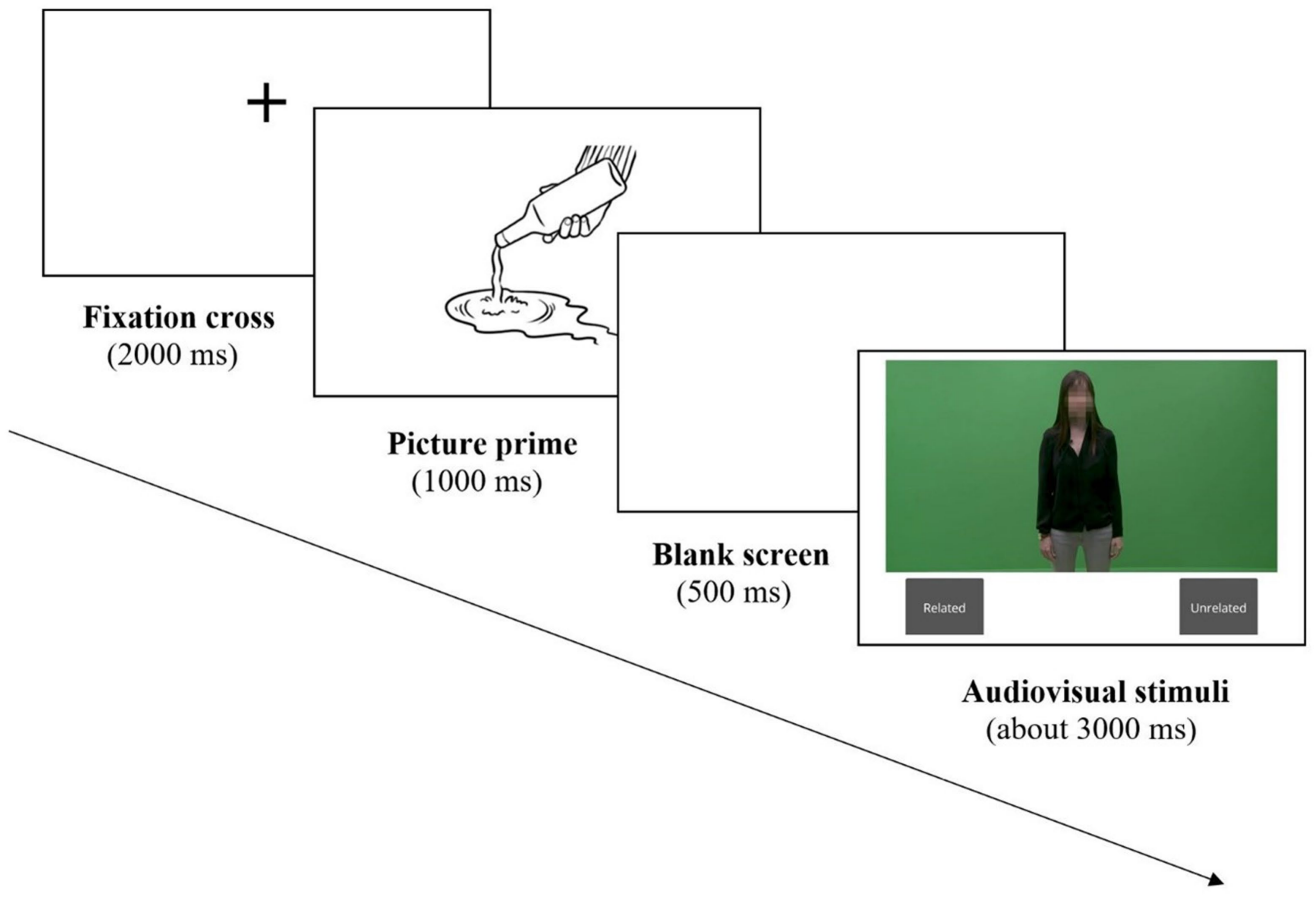
Trial sequence. When the audiovisual stimuli were displayed, participants either viewed them then clicked on the relevant button, “Related” or “Unrelated” (passive viewing/listening activity) or interpreted simultaneously the stimuli then clicked on the relevant button (SI activity).

The stimuli in the experiment were drawings of an action prime (e.g., picking a lemon, petting a cat, or swinging on a rope), followed by a blank screen for 500 ms, and followed by a short video clip with a verbal or gestural target for approximately 3,000 ms (Figure 1). Each video clip was produced in two different conditions: congruent or incongruent speech gesture-pairs.

**Figure 2:**
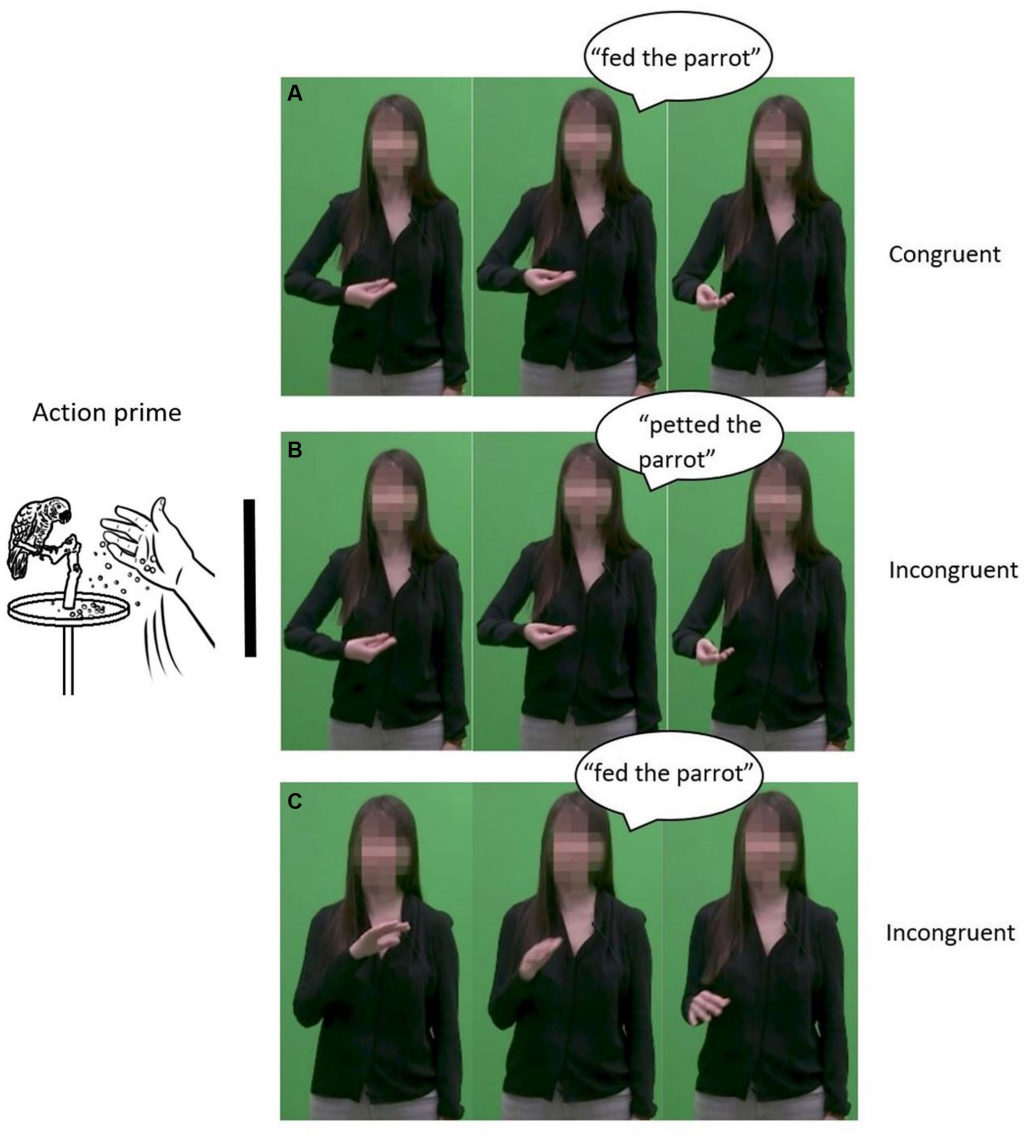
Congruent and incongruent speech-gesture pairs. Panel A presents a congruent speech-gesture pair in which both speech and gesture are related with the prime. Panel B presents an incongruent speech-gesture pair in which speech is unrelated with the prime whereas gesture is related with the prime. Panel B presents an incongruent speechg-gesture pair in which gesture is unrelated with the prime whereas speech is related with the prime.

In the congruent condition, the gesture (e.g., gesturing feeding) and speech (e.g., saying “fed”) conveyed congruent information and both were related to the prime (e.g., feeding a parrot). For the same prime, in the incongruent speech-target condition, the spoken portion of the target was related to the prime, but the gesture was incongruent (e.g., gesturing pet). In the incongruent visual-target condition, the gestural portion of the target was related to the prime, but the speech was incongruent (e.g., saying “petted”). The stimuli were constructed across the two target types (auditory vs. gestural), such that half of the trials had “related” primes and the other half “unrelated.” Figure 2 shows a picture prime illustrating “fed the parrot.” For the speech target, panels A and C are related to the prime, whereas panel B is unrelated. Indeed, in panel A, which displays congruent speech (“fed the parrot”) and gesture (feeding), the auditory part corresponds to the prime. In panel C, in which the auditory of the stimuli (“fed the parrot”) and the gestural portion (petting) are incongruent, the auditory part also corresponds to the prime. Panel B, which includes incongruent speech (“petted the parrot”) and gesture (feeding), is unrelated, however, as the auditory part does not correspond to the prime. Following the same logic, for the visual movement target, panels A and B are related to the prime, whereas panel C is unrelated. Indeed, in congruent panel A (“fed the parrot” with a feeding gesture), the visual (gestural) part corresponds to the prime. In incongruent panel B (“petted the parrot” with a feeding gesture), again, the visual part corresponds to the prime. However, in incongruent panel C (“fed the parrot” with a petting gesture), the visual portion of the stimuli does not correspond to the prime. In addition, one other type of stimulus was presented to create a balanced design. This type of stimulus was both unrelated and incongruent: no portion of the video clip (e.g., neither the auditory nor the gestural part) was related to the prime, and speech and gesture were incongruent. For instance, a video clip where the actress said “petted” and gestured “fed” would be displayed after a prime illustrating “hopped.” All combinations of primes and auditory and gestural stimuli are presented in Table 2.

**Table 2:** Examples of the stimuli in the congruent and incongruent conditions for related and unrelated primes for each half of the participant group: audio targets and visual targets.

Prime		Congruent audio	Congruent visual	Incongruent audio	Incongruent visual
Audio targets					
Related	Fed	“Fed the parrot”	Feeding	“Fed the parrot”	Petting
Unrelated	Hopped	“Fed the parrot”	Feeding	“Fed the parrot”	Petting
Visual targets					
Related	Fed	“Fed the parrot”	Feeding	“Petted the parrot”	Feeding
Unrelated	Hopped	“Fed the parrot”	Feeding	“Petted the parrot”	Feeding

#### 2.2.1. Speech

We created a first set of 28 utterances following one of two patterns: (1) adverbial phrase of time, agent, verb and patient (e.g., “Last Monday, the girl *fed* the parrot.”), or (2) adverbial phrase of time, agent, verb, preposition and indication of location (e.g., “Two weeks ago, the boy *swung on* the rope”). The target word was the main verb. We then created a second set of 28 sentences replacing the verbs with equally plausible candidates (e.g., “Last Monday, the girl *petted* the parrot.” or “Two weeks ago, the boy *climbed up* the rope”). We used those mirrored lists to create congruent and incongruent speech-gesture pairs (see below).

#### 2.2.2. Gestures

We devised manual gestures to accompany the sentences in the congruent condition. Congruent gestures were all representational gestures (following the categorization of gestural functions in Kendon, 2004) corresponding to the content of the target verb. For example, for “fed the parrot,” the speaker performed a gesture in which the right hand was palm facing up with the fingers extended, fingers facing the left-hand side, then did biphasic movements toward the front of the speaker as if throwing something on the ground (cf. Figure 2). Congruent gestures depicted path rather than manner of movement in motion verbs (i.e., they showed a trajectory, e.g., *going up*, but did not provide information about manner of motion, e.g., no wiggling of fingers to indicate climbing).

All 56 sentences were recorded audiovisually by a right-handed female speaker of North-American English in a sound-proof recording studio in controlled lighting conditions. Sentences were read from a prompter. The intended gestural movement for each clip was described to the speaker but she was asked to perform her own version of them so that they would be as natural as possible. All gestures were performed with the speaker’s dominant (right) hand. The mean duration of the audiovisual recordings was 3.05 s (*SD* = 0.32, range: 2.20–3.84).

The speaker’s face was then digitally covered to block access to lip information using Adobe Premiere Pro. Horizontally flipped versions of each video clip were also created with the software, so that the speaker also seemed to be gesturing with her non-dominant hand. Half of all videos thus contained right-handed gestures while the other half of the clips included left-handed gestures. This was to control for a potential right-hand bias.

To create the incongruent conditions, the recorded speech was paired with congruent gestures from other video clips using Adobe Premiere Pro. For example, the incongruent gesture for the stimulus “fed the parrot” was a petting gesture (see Figure 2 for examples of the congruent and incongruent speech-gesture pairs), corresponding to “petted the parrot,” which was the mirror item in the second set of sentences.

A naturalness rating was then conducted to ensure that congruent vs. incongruent gestures were actually perceived as such. Six L1 Swedish speakers took part in the first round, while six L1 Swedish speakers participated in the second round (some raters were involved in both norming rounds). They all declared having a good command of English and had been learning English for 9 years on average (*SD* = 2) in the first round, and for 8 years on average (*SD* = 2) in the second round. A sweepstake incentive of 400 Kr was made available for the first round and of 200 Kr for the second round, which was shorter. Raters were told that they had to rate the naturalness of short *video clips* (gestures were not mentioned). Naturalness was defined as a behaviour that you could come across / that could be expected in real life, whereas artificialness was defined as a behaviour that you would not come across / that would not be expected in real life. Raters were also told that the face of the speaker was blocked to hide her identity and avoid distractions for people who knew her. This was to avoid artificialness ratings due to the speaker’s face not being displayed on the screen. Naturalness was rated on a 6-point Likert-type scale (from 1 = “extremely artificial” to 6 = “extremely natural”). The arithmetic mean being 3.5, we only used incongruent items with a naturalness rating below 3.5 and congruent items with a rating above 3.5 in the experiment. Moreover, the gap between the ratings of a congruent and an incongruent item relating to the same verb had to be more than 1 point for the pair to be used in the experiment. Congruent and incongruent speech-gesture pairs significantly differed with regard to naturalness (congruent: *M* = 4.46, *SD* = 0.59; incongruent: *M* = 1.85, *SD* = 0.54; *p* < 0.001). However, audio and visual targets received comparable naturalness ratings (audio: *M* = 3.11, *SD* = 1.46; visual: *M* = 3.20, *SD* = 1.40).

Gestures were then coded to control for several features to ensure that these were evenly distributed across congruent and incongruent speech-gesture pairs and target types (see Supplementary Appendix S1). All gestures were coded and controlled for viewpoint (character- vs. observer-viewpoint); McNeill (1992). A character-viewpoint gesture incorporates the speaker’s body into gesture space, with the speaker’s hands representing the hands of a character. For example, the speaker may move her hand as if she were slicing meat herself. In contrast, an observer-viewpoint gesture excludes the speaker’s body from gesture space, and hands play the part of a character as a whole. For instance, the speaker may move her hand from left to right with a swinging movement to depict a character swinging on a rope. The congruent and incongruent gesture conditions included the same proportion of character- and observer-viewpoint gestures (57% character viewpoint, 43% observer viewpoint). As to audio and visual targets, they included the same number of character- and observer-viewpoint gestures (57% character-viewpoint, 43% observer-viewpoint).

Gestures were further coded for their timing relative to speech to ascertain that the stroke coincided temporally with the spoken verb form. Verb duration was determined for each video clip by identifying verb onset, offset and preposition offset in the case of the observer-viewpoint category. Mean verb duration was comparable between congruent (*M* = 510 ms, *SD* = 123) and incongruent speech-gesture pairs (*M* = 502 ms, *SD* = 116). Mean verb duration was also comparable between the visual-target (*M* = 503 ms, *SD* = 115) and the audio-target condition (*M* = 509 ms, *SD* = 123).

Stroke duration was determined for all gestures and included post-stroke-holds, when present. Mean stroke duration did not differ significantly between congruent (*M* = 561 ms, *SD* = 119) and incongruent speech-gesture pairs (*M* = 589 ms, *SD* = 125). Mean stroke duration was also comparable between the visual-target (*M* = 561 ms, *SD* = 118) and the audio-target condition (*M* = 589 ms, *SD* = 125).

We further coded gestures for ‘single’ vs. ‘repeated stroke’. In single stroke gestures the stroke is performed once, while in repeated gestures the stroke is repeated twice. The congruent and incongruent conditions were comparable in that regard: the congruent category included 75% single stroke gestures and 25% repeated stroke gestures, while the incongruent category included 73% single stroke gestures and 27% repeated stroke gestures. Target types were also comparable, with audio targets including 73% single stroke gestures and 27% repeated stroke gestures, while visual targets included 75% single stroke gestures and 25% repeated stroke gestures.

Place of gestural articulation was coded following an adapted version of McNeill’s schema of gesture space (McNeill, 1992, p. 89) as in Gullberg and Kita (2009). The ‘center-center’ and ‘center’ categories were merged into one ‘center’ category, while the ‘upper periphery’, ‘lower periphery’, etc., were merged into one ‘periphery’ category. Place of articulation was thus coded as either ‘center’, ‘periphery’ or ‘center-periphery’. Place of articulation was distributed in a similar way across congruent and incongruent pairs: most gestures were articulated centrally (50% for congruent gestures and 55% for incongruent gestures), and in the ‘center-periphery’ area (46% for congruent gestures and 43% for incongruent gestures). 4% (congruent condition) and 2% of gestures (incongruent condition) were articulated in the peripheral area. Audio and visual targets were also similar in terms of place of articulation: most gestures were articulated centrally (55% as compared to 50%), and in the ‘center-periphery’ area (43% as compared to 46%). 2% (audio targets) and 4% (visual targets) of gestures were articulated peripherally.

Gestures were also coded for complexity of trajectory. Straight lines in any direction were coded as a ‘simple trajectory’ and more complex patterns were coded as ‘complex trajectories’ (e.g., when the stroke included a change of direction). Trajectory complexity was comparable between congruent (86% simple, 14% complex trajectory) and incongruent speech-gesture pairs (79% simple, 21% complex trajectory). Trajectory complexity was also similar between the visual-target (86% simple, 14% complex trajectory) and the audio-target condition (79% simple, 21% complex trajectory).

All gestures used in the experiment are described in Supplementary Appendix S2.

#### 2.2.3. Primes

Black-and-white line drawings corresponding to the actions depicted in the target verbs were taken from the IPNP database (Szekely et al., 2004). Since more drawings were needed, most of the pictures were created by an artist using the same format. The drawings were normed for name and concept agreement, familiarity and visual complexity as in Snodgrass and Vanderwart (1980) by 11 L1 English speakers. Pictures that did not yield satisfactory measures were redrawn and normed by 10 L1 English speakers (some raters were involved in both norming rounds). A sweepstake incentive of 50 CHF was made available.

Raters were asked to identify pictures as briefly and unambiguously as possible by typing in the first description (a verb) that came to mind. Concept agreement, which takes into account synonyms (e.g., “cut” and “carve” are acceptable answers for the target “slice”) was calculated as in Snodgrass and Vanderwart (1980). Picture pairs with concept agreement of over 70% were used. The same raters judged the familiarity of each picture, that is, the extent to which they came in contact with or thought about the concept. Concept familiarity was rated on a 5-point Likert-type scale (from 1 = “very unfamiliar” to 5 = “very familiar”). The same raters rated the complexity of each picture, that is the amount of detail or intricacy of the drawings. Picture visual complexity was rated on a 5-point Likert-type scale (from 1 = “very simple” to 5 = “very complex”). The same series of pictures was used in the audio-target as in the visual-target condition, as well as with congruent and incongruent speech-gesture pairs. However, we ensured that related and unrelated pictures had similar characteristics. It was the case for concept agreement (related: *M* = 91%, *SD* = 10 vs. unrelated: *M* = 91%, *SD* = 10), concept familiarity (related: *M* = 3.8, *SD* = 0.6 vs. unrelated: *M* = 3.8, *SD* = 0.6), and visual complexity (related: *M* = 2.9, *SD* = 0.4 vs. unrelated: *M* = 2.9, *SD* = 0.4).

In sum, as shown in Supplementary Appendix S1, target types were balanced in terms of verb duration, gesture viewpoint, stroke type, stroke duration, place of articulation, gesture trajectory, video clip naturalness, concept agreement, concept familiarity, and visual complexity of the picture. Gesture conditions were balanced for verb duration, gesture viewpoint, stroke type, stroke duration, place of articulation, gesture trajectory, concept agreement, concept familiarity, and visual complexity of the picture, but differed significantly (*p* < 0.001) in terms of naturalness.

No rater took part in the experiment.

### 2.3. Procedure

The main task was for participants to relate the picture prime to either the audio or the visual portion of the subsequent video stimuli. Moreover, to ensure that they focused on both the auditory and visual modalities, participants were told that they would take a recall task about the auditory and visual aspects of the videos at the end of the experimental session. This was meant to ensure that participants paid attention to both modalities during the experiment. No recall task was actually administered. Participants were further told that the face of the speaker was hidden in order to avoid distractions for people who might know her.

Half of the participants (*n* = 24) were instructed to focus on the auditory information in the stimuli and to click on one button (“Related”) if the *speech* of the speaker was related to the picture prime, and to click on another button if it was not (“Unrelated”); this was the audio target condition the remaining participants (*n* = 24) were instructed to focus on the visual information in the stimuli and to press one button (“Related”) if the *visual* content of the speaker’s movements was related to the picture prime, and to press another button (“Unrelated”) if it was not; this was the visual target condition.

Moreover, participants were asked to either simultaneously interpret (SI activity) the video content or to watch (passive viewing/listening activity) the audiovisual stimuli. The experiment was administered in English, ensuring that participants would be able to interpret into their L1, Swedish.^3^ All participants engaged in both activities, and activity order was counterbalanced. At the beginning of each activity, participants completed three practice trials.

#### 2.3.1. Passive viewing/listening

Participants were told that they would first see a picture, then a video recording of a short sentence in English that they had to simply watch. They were asked to then click on a button to answer the question whether either auditory or visual information was related to the prime.

#### 2.3.2. Simultaneous interpreting

Participants were told that they would first see a picture, then a video recording of a short sentence in English. They were asked to interpret the video simultaneously into Swedish. Simultaneous interpreting was defined as a spoken simultaneous translation. Participants were asked to try and start interpreting as soon as possible when the video started (to ensure that interpretations would indeed be simultaneous). A video clip not used in the experiment with an overlayed interpretation into Swedish provided by a pilot participant was displayed as an example. Finally, participants were asked to first interpret, then click on a button once they had completed their rendition to answer the question whether either auditory or visual information was related to the prime.

Overall, across both activities, each participant was presented with a randomized sequence of 56 videos twice, once with a related prime, once with an unrelated prime (never in succession), for a total of 112 stimuli. The video clips were paired with 28 picture primes. There were 3 five-minute breaks within the experiment: one halfway of each activity, and one in-between activities. Participants could not force launch the next trials but had to wait until the automatic display of the next screen. A countdown was provided.

For both passive viewing/listening and SI, RT measurement onset was time-locked to the target verb onset in the audio-target condition, and the stroke onset in the visual-target condition. In the SI activity, interpretations were automatically recorded in all trials. Recordings started at the video clip onset and stopped automatically 5 s after the offset of the video clip (this was to give participants enough time to complete their interpretations, while ensuring they would still interpret as simultaneously as possible). If participants clicked on one of the response buttons before then, the recording was stopped.

The study lasted approximately 50 min (including the consent form, the LEAP-Q, the LexTALE, sound and microphone checks, the actual experiment and the debriefing). Participants were paid 9.73 GBP for their participation.

### 2.4. Analysis

We used a mixed design, with Target type (audio vs. visual) as a between-group factor and Audiovisual congruence (congruent vs. incongruent), and Priming relationship (related vs. unrelated) as within-subjects factors.^4^

The analyses for the two dependent variables, response accuracy and reaction time (RT), were conducted separately and implemented in R (R Core Team, 2013) using the lme4 package (Bates et al., 2015). Results from the SI and passive viewing/listening activities were analysed separately.^5^ All data-analysis models are provided in Supplementary Appendix S3.

Practice trials were not included in the analyses. Trials affected by technical problems (e.g., when the platform failed to record participants’ interpretations) were also excluded (34 trials, 0.6% of the whole dataset). Trials in which participants had not interpreted, only partially interpreted, or had not finished interpreting the stimuli by the end of the recording were also excluded from the analysis, which led to the removal of 34.6% of interpreted trials (920 trials, 17.2% of the whole dataset).

#### 2.4.1. Accuracy

Accuracy data was analysed using generalized linear mixed models (GLMM). The dataset was trimmed before completing the analyses. Responses above and below 2 SDs from the RT mean were considered outliers, which led to the removal of 0.9% (8 trials) of the data points in the SI activity dataset, and 3.1% (41 trials) of the passive viewing/listening activity dataset in the audio-target condition. In the visual-target condition, 1.0% (8 trials) of the data points in the SI activity dataset, and 1.6% (21 trials) of the passive viewing/listening activity dataset were removed.

GLMM analyses were conducted on the full dataset to test the relationship between accuracy and the fixed effects *priming relationship* (2 levels, related vs. unrelated prime), *audiovisual congruence* (2 levels, congruent vs. incongruent speech-gesture pair) and *target type* (2 levels, gesture vs. speech). An interaction term was set between priming relationship, audiovisual congruence and target type. Subjects and items were entered as random effects with by-subject and by-item random intercepts as this was the maximal random structure supported by the data. GLMM analyses were also conducted on a subset of related primes only to test the relationship between accuracy and two fixed effects only, namely *audiovisual congruence* (2 levels, congruent vs. incongruent speech-gesture pair) and *target type* (2 levels, gesture vs. speech), with an interaction term. Subjects and items were entered as random effects with by-subject and by-item random intercepts as this was the maximal random structure supported by the data.

#### 2.4.2. Reaction time

Linear mixed-effects model (LMM) analyses were run on the RT data. Significance of effects was determined by assessing whether the associated *t*-statistics had absolute values ≥2. Only accurate trials were used for the RT analyses, which led to the removal of 3.2% (30 trials) of the data points in the SI activity dataset, and 3.7% (50 trials) of the passive viewing/listening activity dataset in the audio-target condition. In the visual-target condition, 21.9% (183 trials) of the data points in the SI activity dataset, and 15.7% (211 trials) of the passive viewing/listening activity dataset were removed. The dataset was trimmed before completing the analyses using the same approach as for the Accuracy data, which led to the removal of another 2.2% (19 trials) of the data points in the SI activity dataset, and 4.5% (58 trials) of the passive viewing/listening activity dataset in the audio-target condition. In the visual-target condition, 1.8% (12 trials) of the RT data points in the SI activity dataset, and 2.9% (33 trials) of the passive viewing/listening activity dataset were removed.

RTs were log-transformed and analysed using a LMM with the same fixed-effects structure as the GLMM. Subjects and items were entered as random effects with by-subject and by-item random intercepts. As for the accuracy analyses, we first probed the full dataset, then investigated the subset of related primes only.

## 3. Results

### 3.1. Accuracy

**Figure 3:**
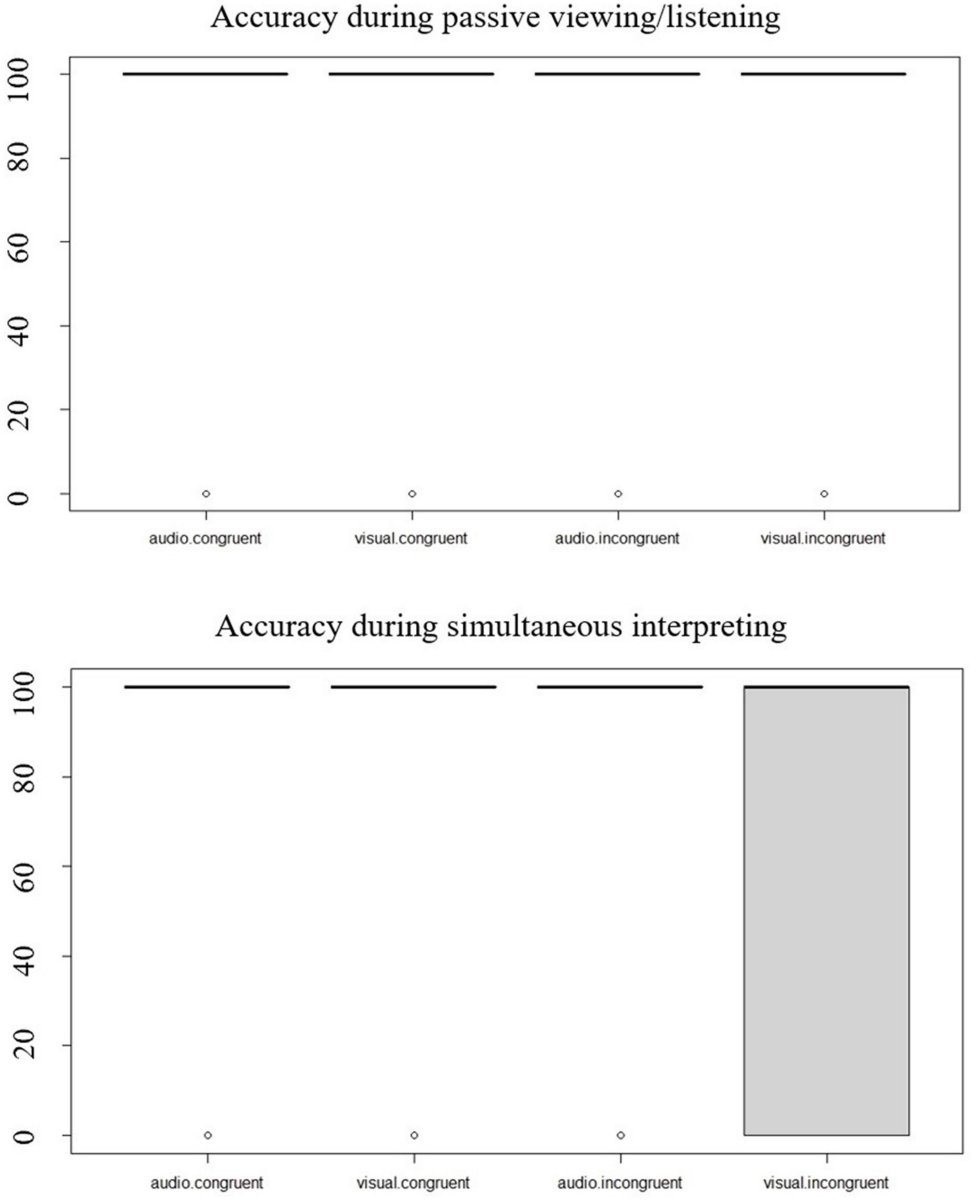
Accuracy in percentages in the passive viewing/listening activity and in the simultaneous interpreting activity according to audiovisual congruence and target type.

Accuracy scores are reported in Table 3A and plotted in Figure 3. Both in the passive viewing/listening and in the SI dataset, visual targets were associated with lower scores, especially in the incongruent condition, whereas Audio targets yielded scores close to ceiling.

**Table 3:** (A) Mean response-accuracy percentages. (B) Mean RT in ms.

Accuracy	Passive viewing/listening		Simultaneous interpreting	
	*M*	*SD*	*M*	*SD*
Audio target				
Congruent pair	97.2	16.6	96.8	17.6
Incongruent pair	96.9	17.5	97.3	16.3
Visual target				
Congruent pair	92.1	27.0	87.4	33.2
Incongruent pair	77.1	42.0	68.0	46.7

RT	Passive viewing/listening		Simultaneous interpreting	
	*M*	*SD*	*M*	*SD*
Audio target				
Congruent pair	1,761	825	4,755	1,240
Incongruent pair	1,844	862	4,858	1,234
Visual target				
Congruent pair	1,697	682	5,191	1,422
Incongruent pair	1,841	794	5,278	1,498

#### 3.1.1. Passive viewing/listening

Looking at the full passive viewing/listening dataset, we used the optimx optimiser (Nash, 2014). The interaction between priming relationship, audiovisual congruence and target type was significant (*β* = 5.22, *SE* = 0.85, *Z* = 6.12, *p* < 0.001), indicating that the three fixed effects interacted to affect accuracy. The result of the likelihood-ratio test used to compare the full to a first reduced model (priming relationship * audiovisual congruence + target type) was significant (*χ*^2^ (3) = 59.27, *p* < 0.001), indicating that the full model was a better fit to the data than the reduced model. The model output indicated that accuracy was significantly affected by an interaction of priming relationship and target type (unrelated prime*visual target: *β* = −1.58, *SE* = 0.61, *Z* = −2.58, *p* = 0.01) and an interaction of audiovisual congruence and target type (incongruent pair*visual target: *β* = −3.64, *SE* = 0.60, *Z* = −6.11, *p* < 0.001). We then fitted an alternative reduced model (priming relationship + audiovisual congruence * target type), and again the result of the likelihood-ratio test used to compare the full model to this reduced model was significant (*χ*^2^ (3) = 151.05, *p* < 0.001), indicating that the full model better fitted the data. Similarly, the third reduced model we fitted (audiovisual congruence + priming relationship * target type) was not as good a fit to the data as the full model (*χ*^2^ (3) = 150.81, *p* < 0.001).

#### 3.1.2. SI

In the full SI dataset, the interaction between priming relationship, audiovisual congruence and target type was significant (*β* = 4.44, *SE* = 0.99, *Z* = 4.50, *p* < 0.001), indicating that the three fixed effects interacted to affect accuracy. The result of the likelihood-ratio test used to compare the full to a first reduced model (priming relationship * audiovisual congruence + target type) was significant too (*χ*^2^ (3) = 36.96, *p* < 0.001), indicating that the full model was a better fit to the data than the reduced model. The model output indicated that accuracy was significantly affected by an interaction of priming relationship and target type (unrelated prime*visual target: *β* = −2.05, *SE* = 0.68, *Z* = −3.03, *p* < 0.01) and an interaction of audiovisual congruence and target type (incongruent pair*visual target: *β* = −3.71, *SE* = 0.63, *Z* = −5.93, *p* < 0.001). We then fitted an alternative reduced model (priming relationship + audiovisual congruence * target type), and again the result of the likelihood-ratio test used to compare the full model to this reduced model was significant (χ^2^ (3) = 108.89, *p* < 0.001), indicating that the full model better fitted the data. Similarly, the third reduced model we fitted (audiovisual congruence + priming relationship * target type) was not as good a fit to the data as the full model (*χ*^2^ (3) = 119.8, *p* < 0.001).

### 3.2. Reaction time

**Figure 4:**
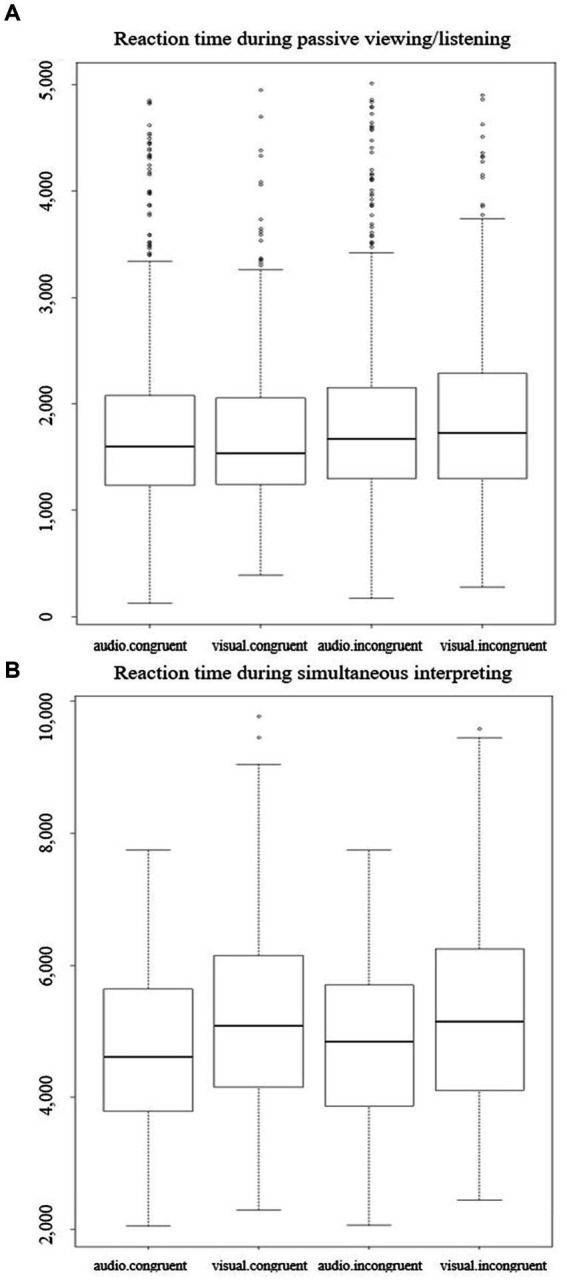
RTs in ms in the passive viewing/listening activity **(A)** and in the simultaneous interpreting activity **(B)** according to audiovisual congruence and target type.

Reaction times are presented in Table 3B and plotted in Figure 4. Across activities and target types, incongruent speech-gesture pairs were associated with longer RTs than congruent pairs. As expected, RT were clearly faster in the passive viewing/listening dataset than in the SI dataset, since participants had to first interpret simultaneously the stimuli before responding in the SI activity. Some recordings included an interpretation, then a silence. In a post-hoc analysis, we set out to ensure that no RT effect was masked by these silences. In each recording, we coded interpretation onset, interpretation duration, the duration of any silence after the interpretation, and the proportion of any final silences in the overall duration of interpretations + silences. We ran the same linear mixed-effects models as on the RT data, but only found interactions including Priming relationship, whereas the variables of interest did not significantly affect any of these measures.

#### 3.2.1. Passive viewing/listening

Looking at the full passive viewing/listening dataset, the interaction between priming relationship, audiovisual congruence and target type was not significant (*β* = −0.01, *SE* = 0.05, *t* = −0.03), indicating that the three fixed effects did not interact to affect RTs. The result of the likelihood-ratio test used to compare the full to a first reduced model (priming relationship + target type * audiovisual congruence) was not significant either (*χ*^2^ (3) = 7.19, *p* = 0.07), confirming this result and indicating that the reduced model was a better fit to the data than the full model. We then fitted an alternative reduced model (priming relationship * target type + audiovisual congruence), and this time the result of the likelihood-ratio test used to compare the full model to this reduced model was significant (*χ*^2^ (3) = 8.29, *p* = 0.04), indicating that the full model better fitted the data. Finally, we fitted a last reduced model (target type + audiovisual congruence * priming relationship), and the result of the likelihood-ratio test used to compare the full model to this reduced model was not significant (*χ*^2^ (3) = 1.73, *p* = 0.63). We then selected the best-fitting reduced model, which was the third reduced model (AIC = 1,411; BIC = 1,457; logLik = −697; full model: AIC = 1,415; BIC = 1,478; logLik = −697; reduced model 1: AIC = 1,416; BIC = 1,462; logLik = −700). The model output indicated that RTs were significantly affected by audiovisual congruence (incongruent pairs: *β* = 0.09, *SE* = 0.02, *t* = 4.76), priming relationship (unrelated prime: *β* = −0.05, *SE* = 0.02, *t* = −2.60) and by an interaction of priming relationship and audiovisual congruence (unrelated prime*incongruent pair: *β* = −0.07, *SE* = 0.03, *t* = −2.57).

#### 3.2.2. SI

In the full SI dataset, the interaction between priming relationship, audiovisual congruence and target type was not significant (*β* = −0.01, *SE* = 0.05, *t* = −0.04), indicating that the three fixed effects did not interact to affect RTs. The result of the likelihood-ratio test used to compare the full to a first reduced model (priming relationship + target type * audiovisual congruence) was not significant either (*χ*^2^ (3) = 7.55, *p* = 0.06), confirming this result. We then fitted an alternative reduced model (priming relationship * target type + audiovisual congruence), and again the result of the likelihood-ratio test used to compare the full model to this reduced model was not significant (*χ*^2^ (3) = 4.39, *p* = 0.22), indicating that the reduced model better fitted the data. Finally, we fitted a last reduced model (target type + audiovisual congruence * priming relationship), and again, the result of the likelihood-ratio test used to compare the full model to this reduced model was not significant (*χ*^2^ (3) = 4.01, *p* = 0.26). We then selected the best-fitting reduced model, which was the third one (AIC = −185; BIC = −142; logLik = 100; vs. reduced model 1: AIC = −181; BIC = −139; logLik = 99; reduced model 2: AIC = −184; BIC = −142; logLik = 100). The output of the best-fitting model indicated that RTs were significantly affected by priming relationship (unrelated prime: *β* = 0.07, *SE* = 0.02, *t* = 4.90) and by audiovisual congruence (incongruent pair: *β* = 0.05, *SE* = 0.02, *t* = 2.94).

## 4. Discussion

This study set out to examine whether manual gestures and speech form a single integrated system during second language comprehension, more specifically in a simultaneous interpreting context, which involves comprehension in one language and simultaneous production in another. First, we set out to replicate previous research showing that incongruent multimodal utterances are harder to process than congruent ones and extend it to L2 comprehenders, especially in a simultaneous interpreting context. Moreover, we wanted to test two predictions: if the Integrated-Systems Hypothesis also applies to L2 comprehension, more specifically to simultaneous interpreting, participants engaged in L2 comprehension should (1) process audio targets as easily as visual targets and (2) have comparable difficulty ignoring, on the one hand, irrelevant gestures when processing speech and, on the other hand, irrelevant speech when processing gestures. The results can be summarized in the following points. Incongruent stimuli led to longer reaction times but did not significantly affect accuracy scores; thus, we only partially replicated the incongruency effect in L2 comprehension. As to our first prediction, there was no effect of target type, demonstrating that L2 comprehenders processed both modalities with comparable speed and accuracy. With regard to our second prediction, although audiovisual congruence and target type did not interact to affect RTs, audiovisual congruence and target type interacted in the accuracy results. More specifically, accuracy was lowest when participants were presented with incongruent speech-gesture pairs in the visual-target condition. In other words, incongruent speech was more disruptive for gesture processing than incongruent gesture was for speech processing in L2 comprehension.

With regard to the incongruency effect, we found no main effect of Audiovisual congruence on response accuracy, meaning that incongruent stimuli were not associated with a higher error rate than congruent stimuli. This result, which applied to both activities, is in contrast with previous results (Kelly et al., 2009, 2015; Özer and Göksun, 2020; Perniss et al., 2020, experiment 4). However, the RT results did align with previous studies, in that incongruent stimuli produced longer RTs than did congruent stimuli in both the audio-target and the visual-target conditions and in both activities. Note that in experiment 3 in Perniss et al. (2020), the incongruence effect was only partially replicated: L1 speakers were less accurate but faster when presented with incongruent speech-gesture pairs compared to with congruent pairs, suggesting a trade-off between accuracy and RT results. The current data does not suggest such a trade-off, but points to a slow-down effect for incongruent compared to congruent speech-gesture pairs, indicating that incongruent pairs were harder to process. To summarize, with regard to incongruency, we partially replicated previous results (Kelly et al., 2009, 2015; Özer and Göksun, 2020; Perniss et al., 2020, experiment 4): incongruent speech-gesture pairs took longer to process (but were *not* associated with a higher error rate) compared to congruent pairs.

Based on the Integrated-Systems Hypothesis we had predicted that, first, audio targets should be processed as easily as visual targets. This was fully confirmed since there was no effect of target type, demonstrating that both modalities were processed with comparable speed and accuracy, in both activities. This matches the results of Kelly et al. (2015), but only the accuracy results of Kelly et al. (2009 – as to the RT data, unexpectedly, participants identified speech targets faster than visual targets).

According to our second prediction, participants should have comparable difficulty ignoring irrelevant information in the two modalities. This was partially confirmed by the RT data, but was not supported by the response accuracy data. Indeed, looking at RTs, there was no interaction between audiovisual congruence and target type. Thus, when one modality was irrelevant to the task, participants were still unable to ignore information conveyed in that modality and, crucially, the effect was comparable across modalities. We thus replicate the results reported in previous studies (Kelly et al., 2009, 2015; Perniss et al., 2020, experiment 4). However, audiovisual congruence and target type interacted in the accuracy results. Accuracy was lowest when participants were presented with incongruent speech-gesture pairs in the visual-target condition. In other words, incongruent speech was more disruptive for gesture processing than incongruent gesture was for speech processing. Thus, it seems easier to ignore incongruent gestures compared to incongruent speech. This is in contrast to previous studies (Kelly et al., 2009, 2015) and to the Integrated-Systems Hypothesis, which predicts no such interaction of congruence and target type. The current study is not the first to report differences between target types, however. Perniss et al. (2020, experiment 4) found that, although incongruent speech and incongruent gesture led to comparable error rates in L1 speakers, incongruent gestures led to higher error rates than incongruent speech in L2 speakers. Second, Özer and Göksun (2020) also reported both longer RTs and lower accuracy with incongruent gesture compared to with incongruent speech in their L1 population. In other words, both studies also reported an interaction, but with an inverse pattern of results compared to the present data.

To summarise, the variables of interest, Audiovisual congruence and Target type yielded robust effects both in the full dataset and in the subset including related primes only^6^. Taken together, the data therefore does not fully support the Integrated-Systems Hypothesis in L2 comprehension, more specifically in simultaneous interpreting. The differences compared to results of previous studies may be explained by the fact that materials differed across studies. First, stimuli length and complexity varied across the three studies. While Kelly et al. (2009), Özer and Göksun (2020), and Perniss et al. (2020) used one verb and/or gesture (e.g., saying or gesturing “chop”), the 2015 study by Kelly and colleagues relied on a verb-object structure (e.g., “drank the coffee), whereas the current study used a more complex structure throughout (e.g., “Last Monday, the girl picked the lemon”). This structure, which was necessary to allow participants to engage in actual simultaneous interpreting, may have created more variation in comprehension compared to the previous studies. Moreover, Kelly et al. (2009) and Özer and Göksun (2020) used one-second videos of actions as primes, while Kelly et al. (2015) relied on written verbs and the present study used line drawings of actions. As stated above, Özer and Göksun (2020) found that incongruent gestures were more disruptive for L1 comprehension than incongruent speech; interestingly, they mentioned the isomorphism of action primes and gestures as one explanation for these results: i.e., it might be more difficult to suppress visual-motoric information than linguistic information, which might have led to this result. Perniss et al. (2020) alone use picture primes (line drawings of objects and actions in experiment 3, and colour photographs of actions in experiment 4).

Picture primes have been used in psychology for various purposes. Most importantly, picture primes have been found to prime actions and target naming just as well as real, tangible objects (Squires et al., 2016; Kithu et al., 2021). Studies investigating semantic processing using masked picture primes have yielded evidence of subliminal priming (Dell'Acqua and Grainger, 1999; Van den Bussche et al., 2009). In a different line of research, ERP studies have revealed that affective and emotional picture primes have an effect on subsequent processing or response to a target (Flaisch et al., 2008; Meng et al., 2012). Therefore, using pictures as primes seems a valid approach.

However, it is possible that the results reported in the present paper are influenced by the relationship between the picture primes and the audiovisual stimuli. The norming study we conducted included naming the actions depicted in the drawings by writing down written verbs. Perhaps it was more difficult to establish a link between picture primes and gestures as compared to between picture primes and verbs, especially in incongruent pairs presented in the visual-target condition, as the meaning of the gesture could not be disambiguated by the spoken verb. Indeed, deriving the meaning of gestures without having access to the accompanying speech can be difficult. For instance, according to Kendon, “gestural actions can only be given a precise interpretation when taken in conjunction with the words associated with them (Kendon, 2004, p. 174); in his transcription system, both speech and gesture are systematically coded (p. 362). Along the same lines, in experimental studies, Krauss et al. (1991) found that deriving the meaning of iconic gestures was difficult in the absence of speech, while Feyereisen et al. (1988) found that gesture classification and interpretation partially depended on information given in speech to reduce polysemy, even though gestural form sometimes sufficed for adequate identification. On the other hand, deriving the meaning of speech without having access to gestures is less of an issue. Therefore, the results we report, especially the significant interaction between Audiovisual congruence and target type affecting response accuracy, might be influenced by this relationship between picture primes and stimuli^7^.

Another difference between the present experiment and previous studies concerns the experimental task. In the current study, participants were assigned to one target type (either visual or auditory). Therefore, they were focusing on one target type to judge whether it was related to the prime, or not. That said, they had to pay attention to both gestures and speech, as they were told that they would have to complete a recall task at the end of the experimental session. This design was inspired by the original study by Kelly et al. (2015), but it is slightly different from the design adopted in Kelly et al. (2009, experiment 1); Özer and Göksun (2020); Perniss et al. (2020, experiment 4). Indeed, in these experiments, participants had to press a button if *no part* of the video (speech or gesture) was related to the prime, and another if *any part* of the video (speech or gesture) was related to the prime. Therefore, it seems that participants had to process both modalities more directly to be able to complete the task, whereas the design adopted in the current paper focused on one modality, with an indication to also take the other into account for a later task. Furthermore, in Kelly et al. (2009, experiment 2); Perniss et al. (2020, experiment 3), the task consisted in judging whether the speech content in the video was the same or different from the prime. In other words, participants could pay attention to speech only to complete the task, which is not entirely comparable either. These differences in the design of the experimental task may have influenced the results.

The proficiency level of L2 comprehenders is another factor which may explain the differences between the current data and the study reported in Perniss et al. (2020, experiment 4). In the latter study, L2 comprehenders rated their English proficiency as 4.03 out of 5 (with 1 = “not very good” and 5 = near native”), and were first exposed to English at 9.6 years of age (range 1–23). Unfortunately, those self-reported measures are the only one included in Perniss et al. (2020). The L2 comprehenders in the current paper rated their level of English proficiency as 9.0 out of 10 (*SD* = 1.0) with 1 = “none” and 10 “perfect.” They were first exposed to English at 8.1 years of age (range 1–19). Although they were able to engage in simultaneous interpreting, suggesting an advanced level in their L2, which was corroborated by their LexTALE score, it is difficult to compare them to the group tested by Perniss and colleagues in the absence of objective language measures. It remains that the proficiency level might explain the differences in the results, as different groups of L2 speakers have been shown to benefit differently from visual cues, including gestures, in comprehension tasks (e.g., Sueyoshi and Hardison, 2005).

Despite these differences pertaining to the materials, experimental tasks and language profiles, what clearly comes out of the analyses is that SI and passive viewing/listening are comparable across the board. Indeed, the data revealed the same effects and interactions with regard to the variables of interest (although RTs were longer during SI as compared to passive viewing/listening, due to the design of the experiment). With an incongruency effect affecting RTs in both modalities, equally easy processing of audio and visual targets, and comparable slow-down effects of incongruent speech and incongruent gestures, the data suggests that gestures are part of language comprehension in L2 contexts, even in extreme instance such as SI. SI is considered mentally taxing (Seeber, 2015), as simultaneous interpreters produce a verbal response while comprehending the speaker’s input, but that does not seem to modulate the mutual influence of gesture and speech in language processing. Therefore, the language comprehension component in SI seems to share features similar to other (L2) comprehension tasks. This is in line with the results reported in Arbona et al. (2023) which revealed that multimodal language processing facilitates comprehension in SI the same way as in language comprehension. SI has traditionally been considered and modelled as a first and foremost verbal task (Seeber, 2017), and these results strengthen the emerging case (Galvão and Galhano Rodrigues, 2010; Seeber, 2017; Stachowiak-Szymczak, 2019) for SI to be considered a multimodal phenomenon. As such, they call for models of SI to better take into account the multimodal nature of the task.

### 4.1. Conclusion

The present study investigated whether speech and gesture mutually and obligatorily interact with each other during L2 language comprehension, more specifically, in a simultaneous interpreting context. Incongruent speech-gesture pairs slowed down processing, pointing to an obligatory interaction of gesture and speech. However, incongruent speech was more disruptive for gesture processing than incongruent gesture was for speech processing, which goes against the prediction of mutual interaction. Therefore, the data only partially supports the Integrated-Systems Hypothesis in L2 comprehension. It seems that in (advanced) L2 comprehenders, manual gestures are not completely on an equal footing with speech. More research is needed, however, to better understand how L2 comprehension may differ from L1 comprehension in that regard. That said, we draw two important conclusions from this study. One important result is that the effect of manual gestures on comprehension was not modulated by the SI activity, since “pure” L2 comprehension and SI yielded comparable results. This suggests that the language comprehension component in SI shares common features with other (L2) comprehension tasks. Second, even though it seems that manual gestures and speech are not entirely equal partners in L2 comprehension, gestures do influence L2 comprehension, not least during SI, a case of extreme L2 use.

## Data availability statement

The data that support the findings of this study is available at: https://doi.org/10.17605/OSF.IO/8FG5N.

## Ethics statement

The studies involving human participants were reviewed and approved by Ethics committee of the Faculty of Translation and Interpreting of the University of Geneva. The patients/participants provided their written informed consent to participate in this study.

## Author contributions

EA coded the study, collected the data, performed the statistical analysis, and wrote the first draft of the manuscript. All authors contributed to conception and design of the study, manuscript revision, read, and approved the submitted version.

## Funding

This research was funded by the Swiss National Research Foundation (grant number: P1GEP1_199618).

## Conflict of interest

The authors declare that the research was conducted in the absence of any commercial or financial relationships that could be construed as a potential conflict of interest.

## Publisher’s note

All claims expressed in this article are solely those of the authors and do not necessarily represent those of their affiliated organizations, or those of the publisher, the editors and the reviewers. Any product that may be evaluated in this article, or claim that may be made by its manufacturer, is not guaranteed or endorsed by the publisher.

## Footnotes

^1^www.prolific.co

^2^www.gorilla.sc

^3^Professional interpreters usually work into their L1, and we applied this principle here, which was all the more important given that they had no prior interpreting experience.

^4^We did not predict any differences between “related” and “unrelated” primes, but included both conditions in the analysis to increase power. Analyses of the subset of “related” primes revealed the same effects as in the full dataset with respect to Audiovisual congruence and Target type.

^5^We did not directly compare passive viewing/listening and SI, since the RT data would not have been comparable anyway due to the margin given for the interpretation. Rather, we compared the results of the statistical analyses in each activity subset.

^6^Please note that the analyses yielded a series of effects of priming or of interactions including priming in both accuracy and RTs in both activities. Kelly et al. (2015) The processing of speech, gesture, and action during language comprehension. *Psychon. Bull. Rev.*, 22 (2), 517–523. doi: 10.3758/s13423-014-0681-7 also found contrasting effects of priming (including in interactions with other variables). For example, related trials produced more errors and faster RTs than did unrelated trials; and congruent stimuli produced fewer errors than incongruent stimuli in the related condition, but incongruent stimuli produced fewer errors than congruent stimuli in the unrelated condition. The 2009 study by Kelly et al. included analyses of items with related primes only (items with unrelated primes served as filler items) and therefore yielded no results in terms of priming. Since priming was not the main variable of interest, we do not further discuss these results.

^7^Before conducting the actual experiment, we ran two pilot studies, one using picture primes as in the current experiment (*n* = 4), and another one using written verbs as primes (*n* = 4). As we set out to investigate simultaneous interpreting, a task thought to rely mainly on verbal (=oral) resources, however, we decided to use pictures rather than written verbs as primes so as not to create an imbalance between the auditory and the visual modality.
