# Supplementary material for: The role of manual gestures in second language comprehension: a simultaneous interpreting experiment
Source: Front Psychol. 2023 Jun 27;14:1188628. doi: 10.3389/fpsyg.2023.1188628 (PMC10333536; doi:10.3389/fpsyg.2023.1188628)
Supplement: Supplementary file 1 [file Table_1.DOCX]

S1. Characteristics of the stimuli

Table 1. *Lists and comparison of target types (t-test for numerical variables, Wilcoxon test for ordinal variables): *: p < 0.05, **: p < 0.01, *** : p < .001.*

|  | | **Audio** | | | | **Visual** | | | **Comparison** |
| --- | --- | --- | --- | --- | --- | --- | --- | --- | --- |
|  | | *M* | | *SD* | | *M* | *SD* | |  |
| ***Sentence criteria*** | | | | | | | | | |
| Target verb duration *(ms)* | | 509 | | 123 | | 503 | 115 | | *(ns)* |
| ***Gesture criteria*** | | | | | | | | | |
| Viewpoint *(%)* | | 57 character viewpoint  (32 items)  43 observer viewpoint  (24 items) | | | | 57 character viewpoint  (32 items)  43 observer viewpoint  (24 items) | | |  |
| Stroke type *(%)* | | 73 single (41 items)  27 repeated (15 items) | | | | 75 single (42 items)  25 repeated (14 items) | | |  |
| Stroke duration including post-stroke holds *(ms)* | | 589 | | 125 | | 561 | 118 | | *(ns)* |
| Place of articulation *(%)* | | 55 center (31 items)  2 periphery (1 item)  43 periphery-center  (24 items) | | | | 50 center (28 items)  4 periphery (2 items)  46 periphery-center  (26 items) | | |  |
| Trajectory *(%)* | | 79 simple (44 items)  21 complex (12 items) | | | | 86 simple (48 items)  14 complex (8 items) | | |  |
| ***Video criteria*** | | | | | | | | | |
| Naturalness | 3.11 | | 1.46 | | 3.20 | | 1.40 | *(ns)* | |

Table 2. *Gesture conditions and comparison of gesture conditions (t-test for numerical variables): *: p < 0.05, **: p < 0.01, *** : p < .001.*

|  | **Congruent gestures** | | **Incongruent gestures** | | | | |  | **Comparison** |  |
| --- | --- | --- | --- | --- | --- | --- | --- | --- | --- | --- |
|  | *M* | *SD* | *M* | *SD* | |  |  | |  |  |
| ***Sentence criteria*** | |  |  |  | |  |  | |  |  |
| Target verb duration *(ms)* | 510 | 123 | 502 | 116 | |  |  | | *(ns)* |  |
| ***Gesture criteria*** |  |  |  |  | |  |  | |  |  |
| Viewpoint *(%)* | 57 character viewpoint  (16 items)  43 observer viewpoint  (12 items) | | 57 character viewpoint  (32 items)  43 observer viewpoint  (24 items) | | | |  | |  |  |
| Stroke type *(%)* | 75 single (21 items)  25 repeated (7 items) | | 73 single (41 items)  27 repeated (15 items) | | | |  | |  |  |
| Stroke duration including post-stroke holds *(ms)* | 561 | 119 | 589 | 125 | |  |  | | *(ns)* |  |
| Place of articulation *(%)* | 50 center (14 items)  4 periphery (1 item)  46 periphery-center (13 items) | | 55 center (31 items)  2 periphery (1 item)  43 periphery-center (24 items) | | | | | |  |  |
| Trajectory *(%)* | 86 simple  (24 items)  14 complex  (4 items) | | 79 simple  (44 items)  21 complex  (12 items) | | |  |  | |  |  |
| ***Video criteria*** | | | | | | | | | | |
| Naturalness | 4.46 | 0.59 | 1.85 | | 0.54 | | |  | ***** | |

Table 3. *Priming relationship and comparison of picture primes (t-test for numerical variables): *: p < 0.05, **: p < 0.01, *** : p < .001.*

|  | **Unrelated** | | **Related** | |  | | **Comparison** | | |
| --- | --- | --- | --- | --- | --- | --- | --- | --- | --- |
|  | *M* | *SD* | *M* | *SD* | |  | |  |  |
| ***Picture criteria*** | |  |  |  | |  | |  |  |
| Concept agreement (%) | 91 | 10 | 91 | 10 | |  | |  | *(ns)* |
| Concept familiarity | 3.8 | 0.6 | 3.8 | 0.6 | |  | |  | *(ns)* |
| Visual complexity | 2.9 | 0.4 | 2.9 | 0.4 | |  | |  | *(ns)* |
