# Supplementary material for: The role of manual gestures in second language comprehension: a simultaneous interpreting experiment
Source: Front Psychol. 2023 Jun 27;14:1188628. doi: 10.3389/fpsyg.2023.1188628 (PMC10333536; doi:10.3389/fpsyg.2023.1188628)
Supplement: Supplementary file 3 [file Table_3.DOCX]

**Data-analysis models**

GLMMs: family = binomial, link = "logit"

LMMs: REML = FALSE

All models dummy-coded

*Full passive viewing/listening dataset + full simultaneous interpreting dataset (analyses run separately)*

GLMM accuracy analysis (glmer function, optimizer: optimx/nlminb):

Full model: Accuracy ~ Priming_relationship * Audiovisual_congruence * Target_type + (1|Subject) + (1|Item)

Reduced models: Accuracy ~ Priming_relationship + Audiovisual_congruence * Target_type + (1|Subject) + (1|Item)

Accuracy ~ Priming_relationship * Audiovisual_congruence + Target_type + (1|Subject) + (1|Item)

Accuracy ~ Priming_relationship * Target_type + Audiovisual_congruence (1|Subject) + (1|Item)

LMM RT analysis (lmer function):

Full model: LogRT ~ Priming_relationship * Audiovisual_congruence * Target_type + (1|Subject) + (1|Item)

Reduced models: LogRT ~ Priming_relationship + Audiovisual_congruence * Target_type + (1|Subject) + (1|Item)

LogRT ~ Priming_relationship * Audiovisual_congruence + Target_type + (1|Subject) + (1|Item)

LogRT ~ Priming_relationship * Target_type + Audiovisual_congruence (1|Subject) + (1|Item)

*Related primes subsets – analyses run separately for passive viewing/listening and simultaneous interpreting*

GLMM accuracy analysis (glmer function):

Full model: Accuracy ~ Audiovisual_congruence * Target_type + (1|Subject) + (1|Item)

Reduced model: Accuracy ~ Audiovisual_congruence + Target_type + (1|Subject) + (1|Item)

LMM RT analysis (lmer function):

Full model: LogRT ~ Audiovisual_congruence * Target_type + (1|Subject) + (1|Item)

Reduced model: LogRT ~ Audiovisual_congruence + Target_type + (1|Subject) + (1|Item)
